# Supplementary material for: Molecular weight fractionation by confinement of polymer in one-dimensional pillar[5]arene channels
Source: Nat Commun. 2019 Jan 29;10:479. doi: 10.1038/s41467-019-08372-1 (PMC6351637; doi:10.1038/s41467-019-08372-1)
Supplement: Supplementary file 1 — Supplementary Information [file 41467_2019_8372_MOESM1_ESM.pdf]

Molecular weight fractionation by confinement of polymer in one-dimensional pillar[5]arene channels

Ogoshi et al.

	Water	Acetonitrile
Time (min.)	A(%)	B(%)
0.0	85	15
10	65	35
20.0	0	100
27.5	0	100
27.5	85	15
35.0	85	15

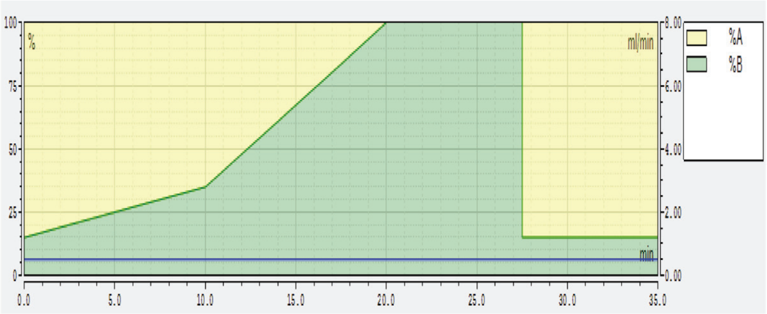

Supplementary Fig. 1 Gradient program of eluents for investigation on molecular weight distribution of PEO.

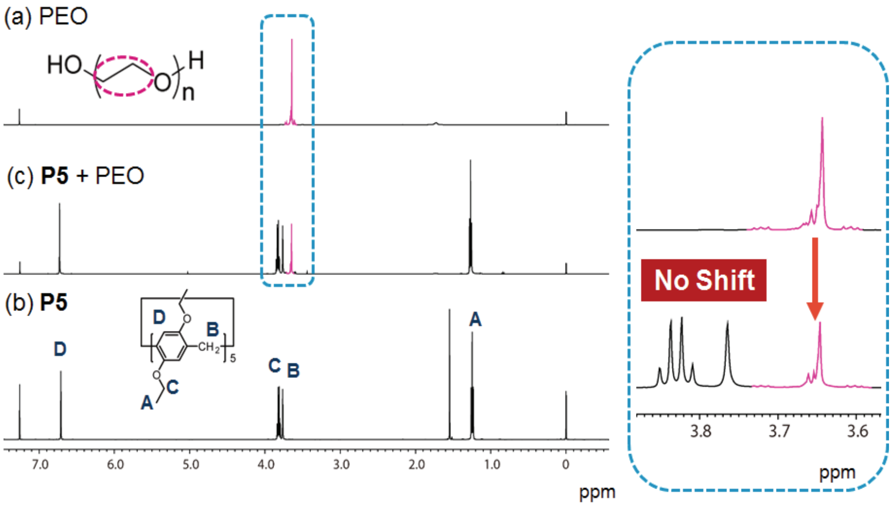

Supplementary Fig. 2 ^{1}H NMR spectra ($CDCl_3$, 25 ^oC) of (a) PEO1000-OH, (b) **P5** and (c) a mixture of PEO1000-OH (58 mM) and **P5** (19 mM).

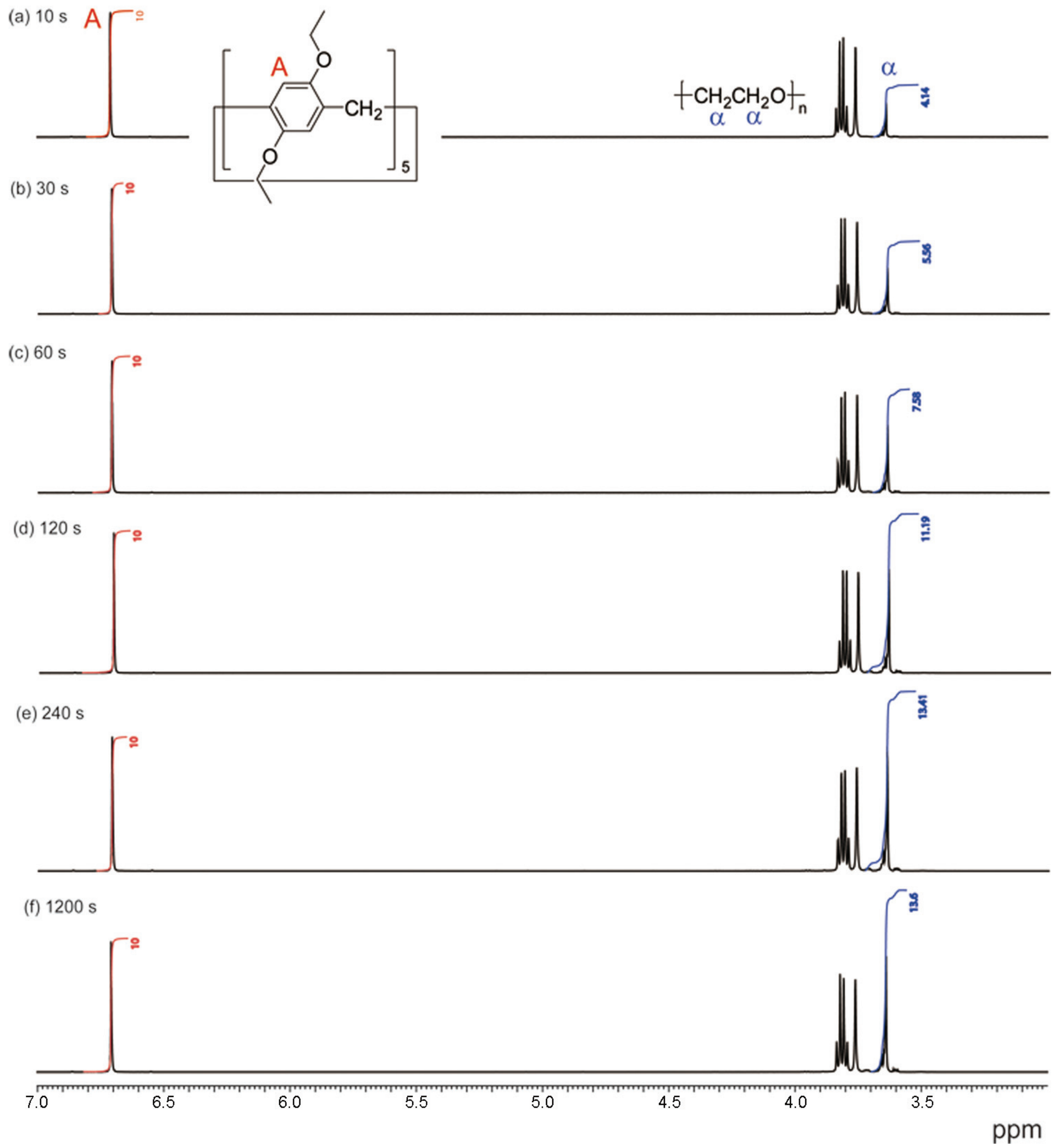

Supplementary Fig. 3 ^{1}H NMR spectra of activated pillar[5]arene crystals **P5** after immersing in melted PEO1000-OH for (a) 10 s, (b) 30 s, (c) 60 s, (d) 120 s, (e) 240 s and (f) 1200 s.

Supplementary Note 1 Increasing integration of the proton signals of PEO by increasing the immersing time indicates PEO uptake by immersing crystals **P5** in melted PEO.

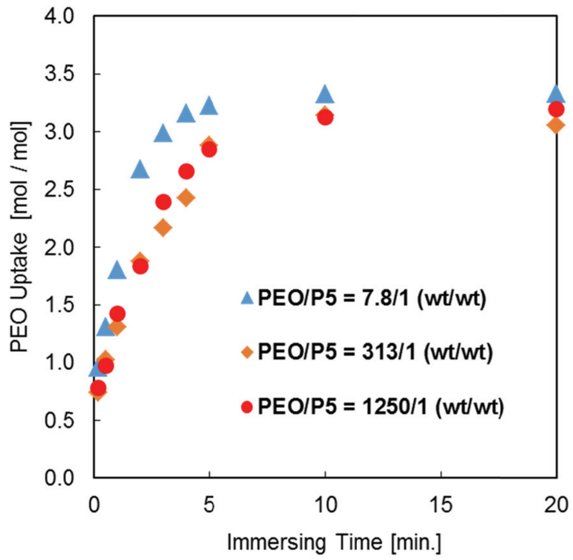

Supplementary Fig. 4 Effect of PEO/**P5** feed ratios on the uptake time and the uptake ratio at equilibrium state. PEO1000-OH and activated crystals of **P5** were used.

Supplementary Note 2 The uptake time and the uptake ratio at equilibrium state were independent within the feed ratios. Therefore, we performed the PEO uptake experiment within the feed ratios.

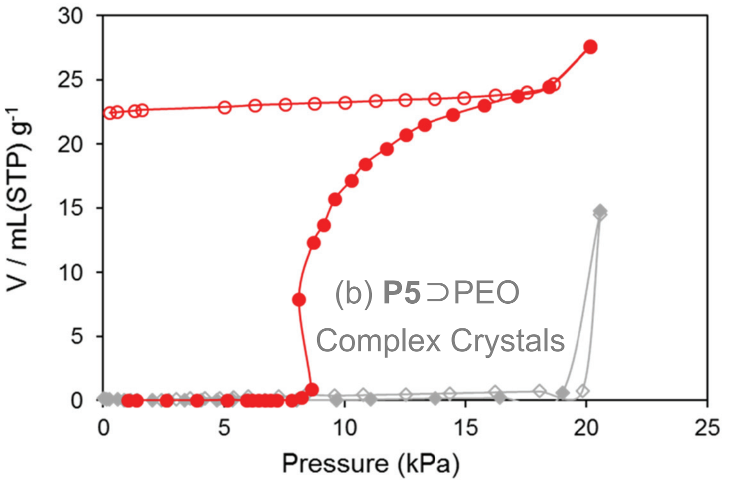

Supplementary Fig. 5 *n*-Hexane vapor sorption isotherms of (a) activated crystals of **P5** (red circles) and (b) **P5**⊃PEO host-guest complex crystals (gray diamonds). Solid symbols: adsorption; open symbols: desorption.

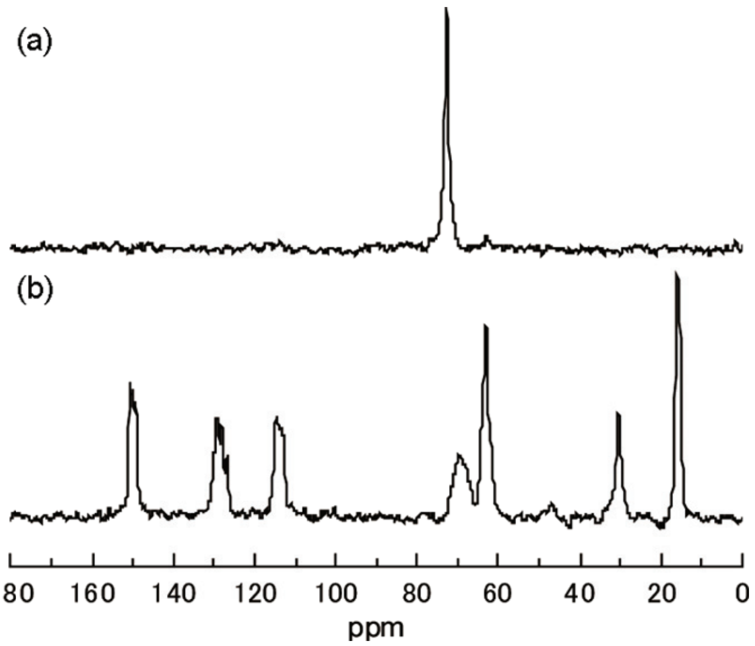

Supplementary Fig. 6 Full scale solid state ^{13}C NMR spectra (Fig. 2c) of (a) neat PEO and (b) **P5**⊃PEO host-guest complex crystals.

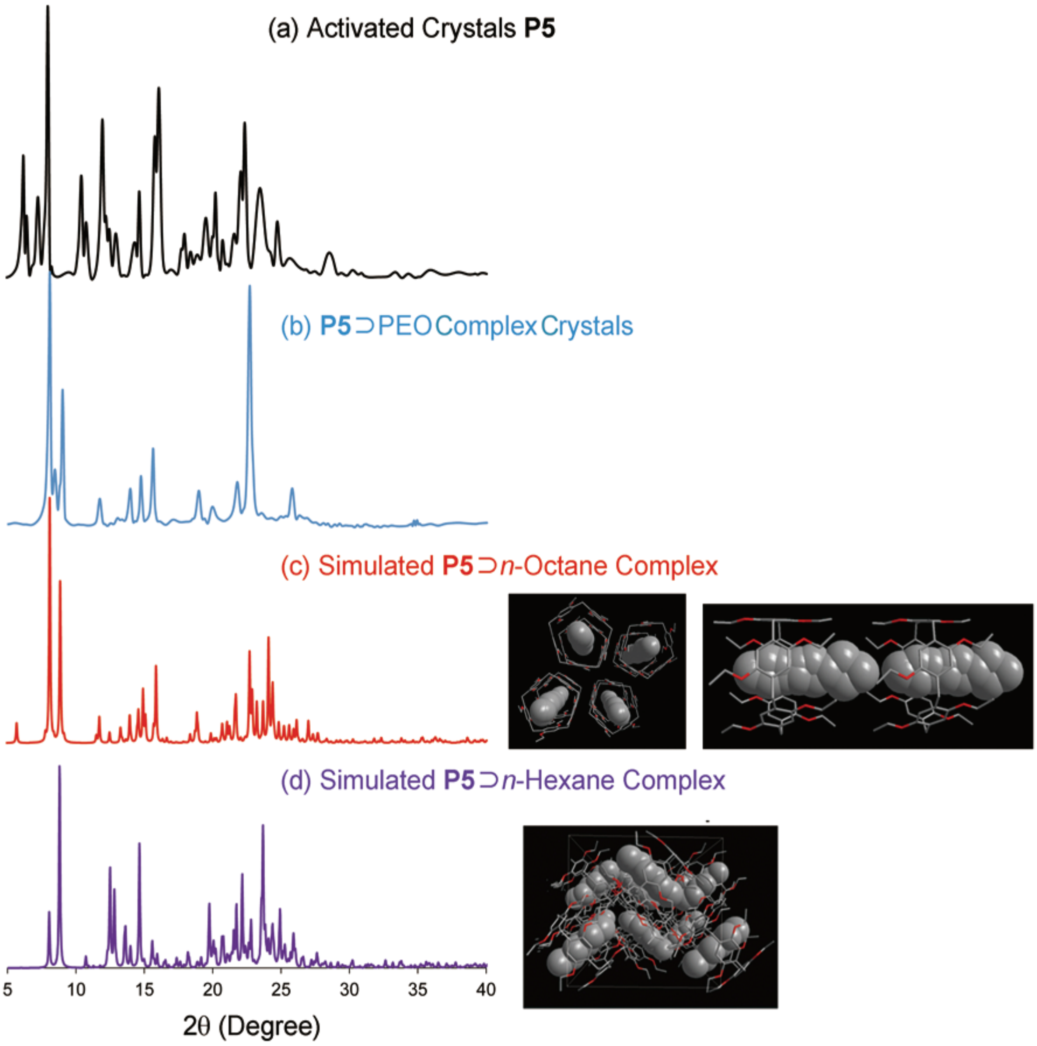

Supplementary Fig. 7 Powder X-ray diffraction patterns of (a) activated crystals of **P5** (black line) and (b) host-guest complex crystals of **P5**⊃PEO complex (blue line). The simulated PXRD patterns determined from the single X-ray crystal structures of (c) **P5** ⊃*n*-octane host-guest complex (red line) and (d) **P5**⊃*n*-hexane host-guest complex (purple line).

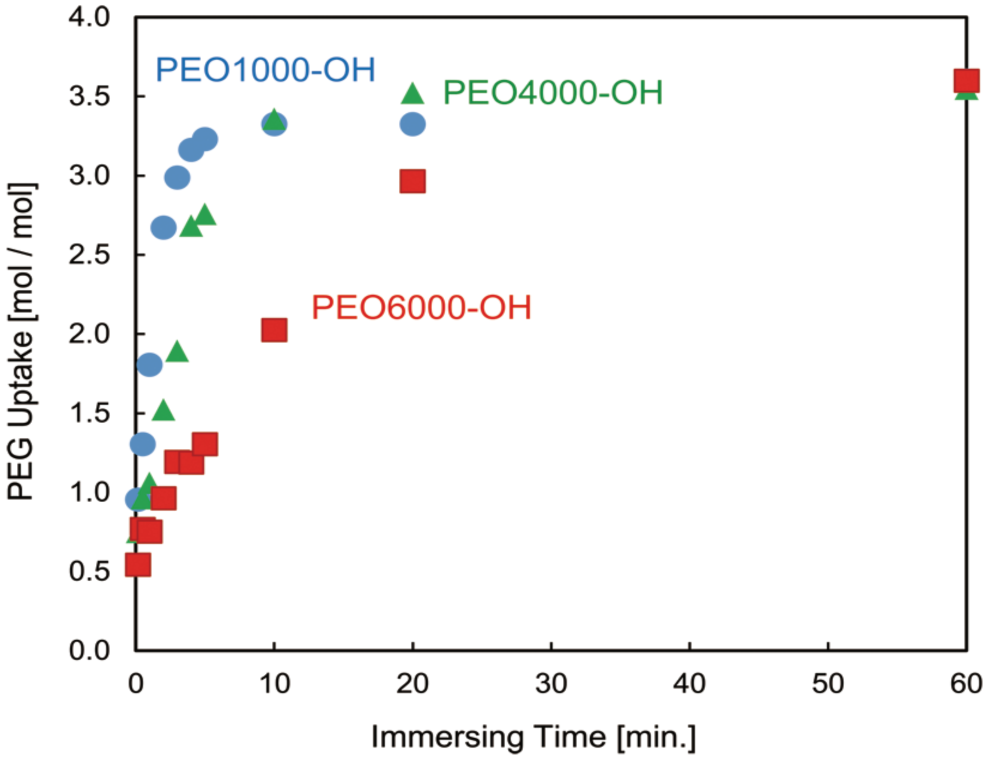

Supplementary Fig. 8 Effect of molecular weight of PEO on the uptake time and the uptake ratio at equilibrium state. PEO with OH ends [PEO1000-OH (blue circles), PEO4000-OH (green triangles) and PEO6000-OH (red squares)] and activated **P5** crystals were used.

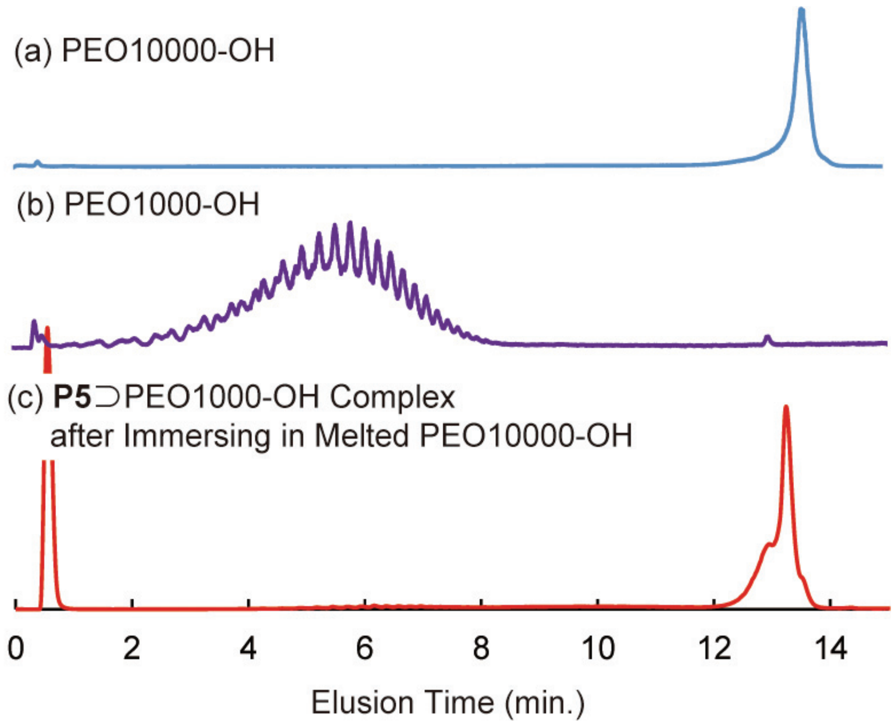

Supplementary Fig. 9 Liquid Chromatograms of (a) PEO1000-OH, (b) PEO10000-OH and (c) **P5**⊃PEO1000-OH complex crystals after immersing to melted PEO10000-OH.

Supplementary Note 3 The peak from PEO10000-OH was observed (Fig. S9c), but not from PEO1000-OH, indicating PEO exchange occurred from PEO1000-OH to PEO10000-OH.

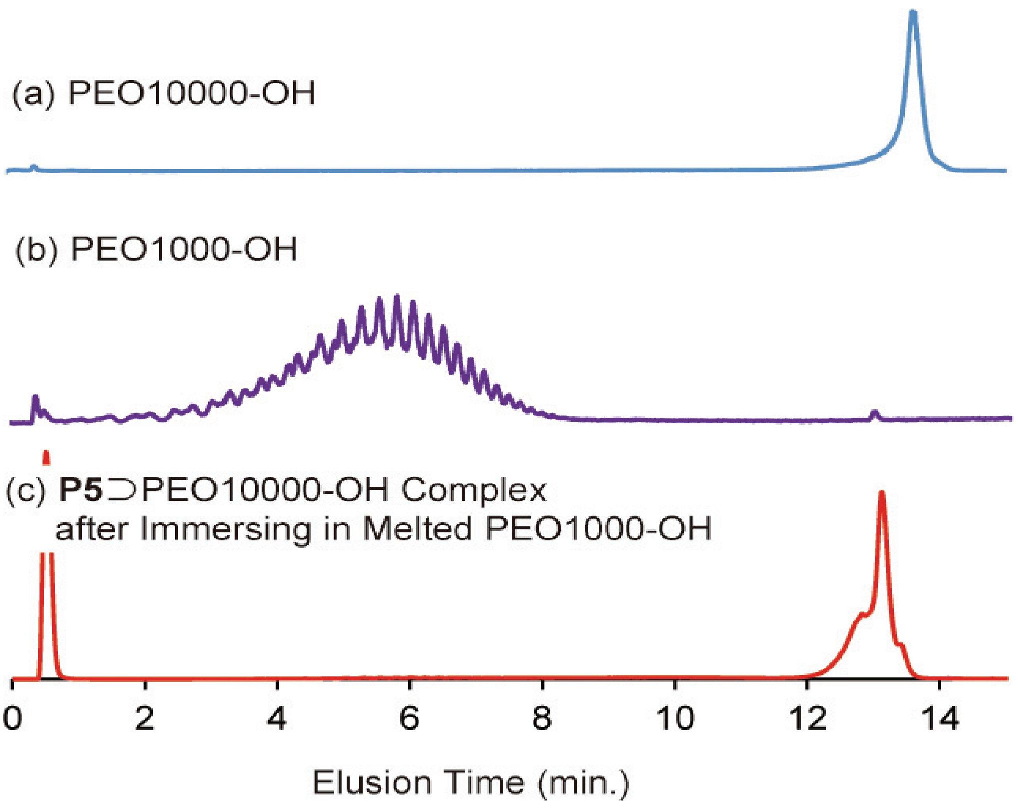

Supplementary Fig. 10 Liquid Chromatograms of (a) PEO1000-OH, (b) PEO10000-OH and (c) **P5**⊃PEO10000-OH complex crystals after immersing to melted PEO1000-OH.

Supplementary Note 4 The peak from PEO10000-OH was observed (Fig. S10c), but not from PEO1000-OH, indicating that guest exchange was negligible.

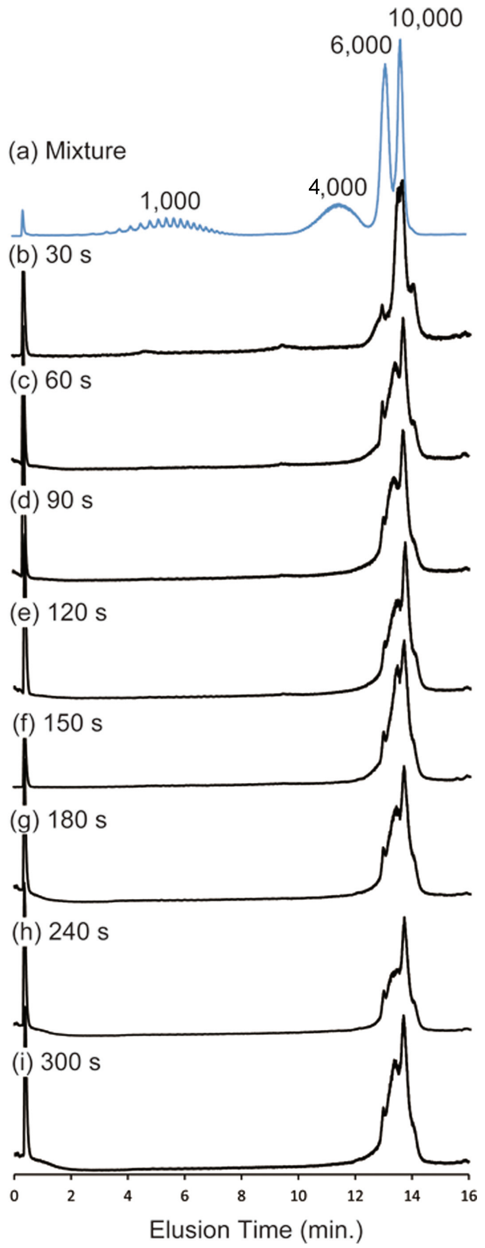

Supplementary Fig. 11 Liquid Chromatograms of (a) an equal-weight mixture of PEO (PEO1000-OH, PEO4000-OH, PEO6000-OH and PEO10000-OH) and activated crystals of **P5** after immersing in the equal-weight mixture of melted PEO at 80 ^{o}C for (b) 30, (c) 60, (d) 90, (e) 120, (f) 150, (g) 180, (h) 240 and (i) 300 s.

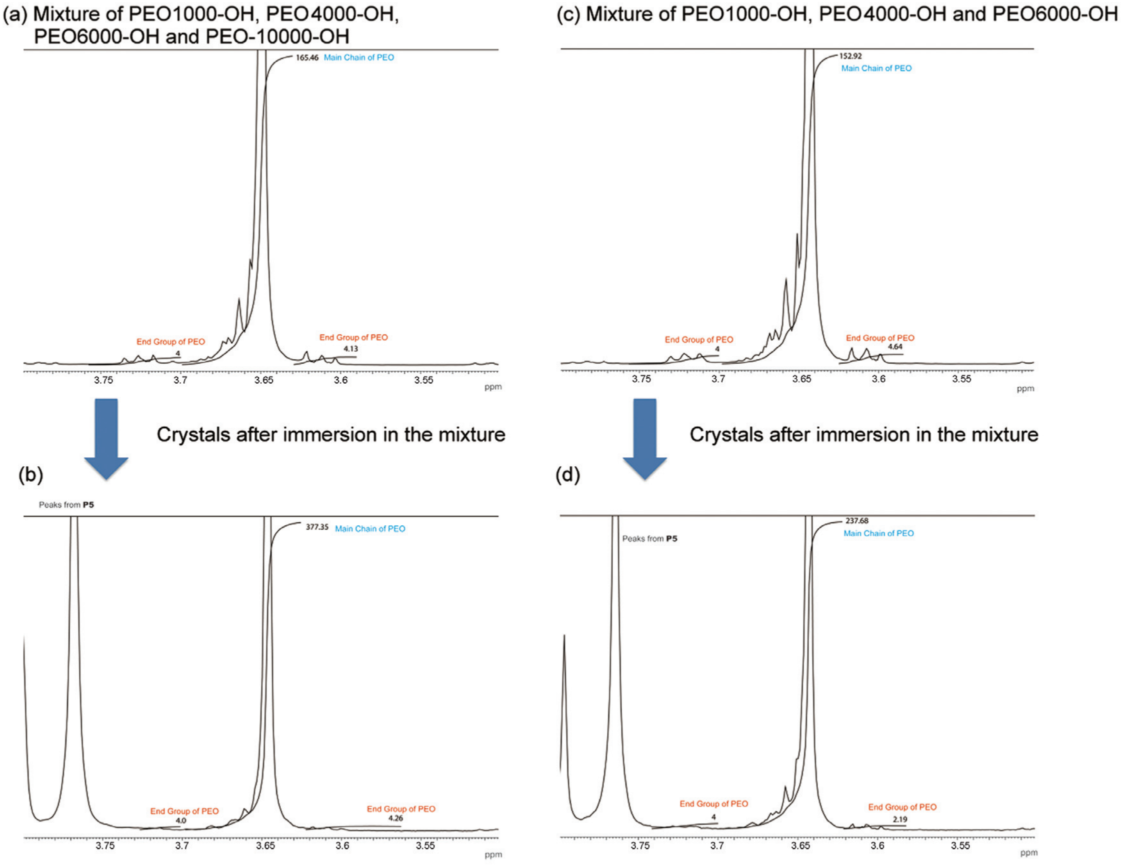

Supplementary Fig. 12 ^{1}H NMR spectra ($CDCl_3$, 25 ^{o}C) of an equal-weight mixture of (a) PEO (PEO1000-OH, PEO4000-OH, PEO6000-OH and PEO10000-OH) and (c) PEO (PEO1000-OH, PEO4000-OH and PEO6000-OH), host-guest complex crystals of **P5** after immersing in the equal-weight mixture of (b) melted PEO (PEO1000-OH, PEO4000-OH, PEO6000-OH and PEO10000-OH) and (d) melted PEO (PEO1000-OH, PEO4000-OH and PEO6000-OH).

Supplementary Note 5 After immersing activated **P5** crystals in the equal-weight mixture of PEO, the integration ratios (main chain of PEO / end group of PEO) were increased, indicating that **P5** crystals took up high mass PEO fraction.

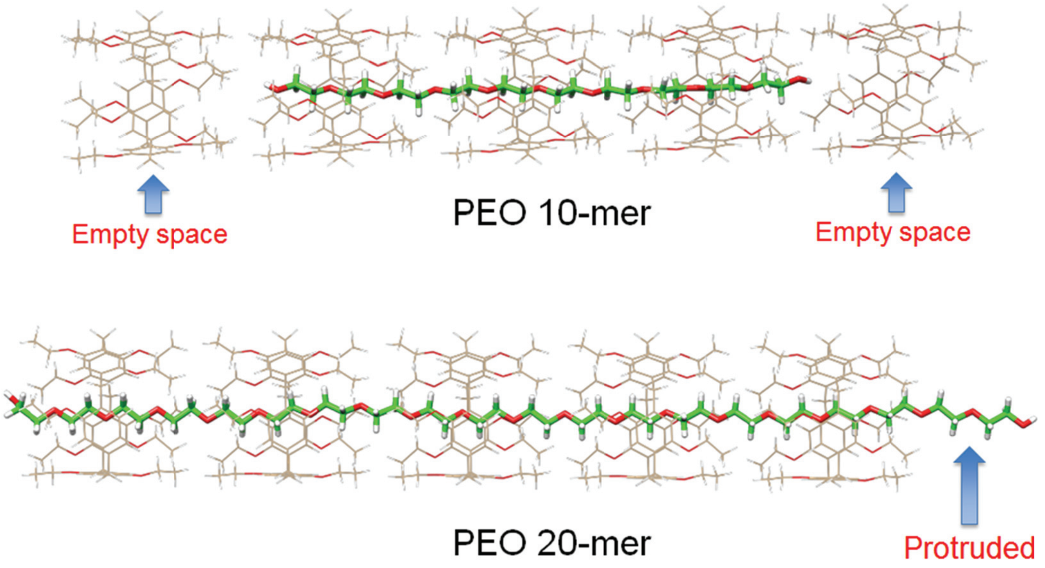

Supplementary Fig. 13 Optimized structures of five **P5**⊃PEO 10-mer or PEO 20-mer. PEO 10-mer is covered by five **P5**, whereas PEO 20-mer is not covered by five **P5**.

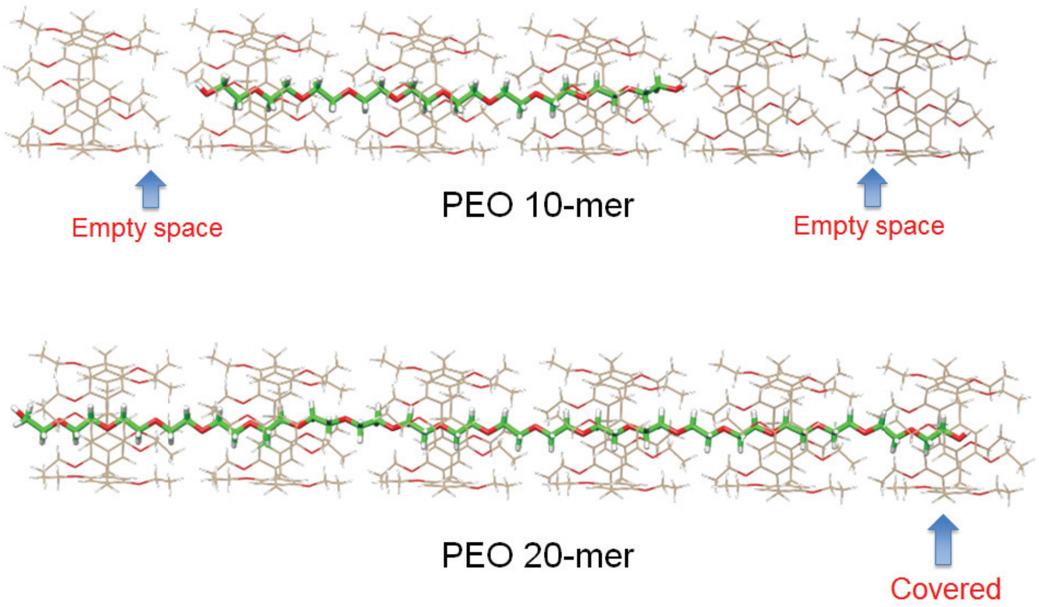

Supplementary Fig. 14 Optimized structures of six **P5**⊃PEO 10-mer or PEO 20-mer. PEO 10-mer is covered by six **P5**. PEO 20-mer is almost covered by six **P5**.

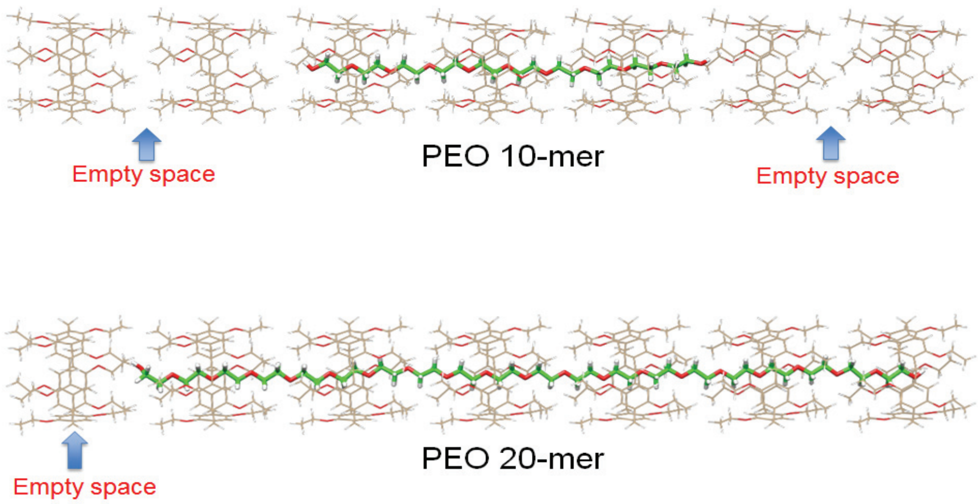

Supplementary Fig. 15 Optimized structures of seven **P5**⊃PEO 10-mer or PEO 20-mer. PEO 10-mer or PEO 20-mer is covered by seven **P5**.

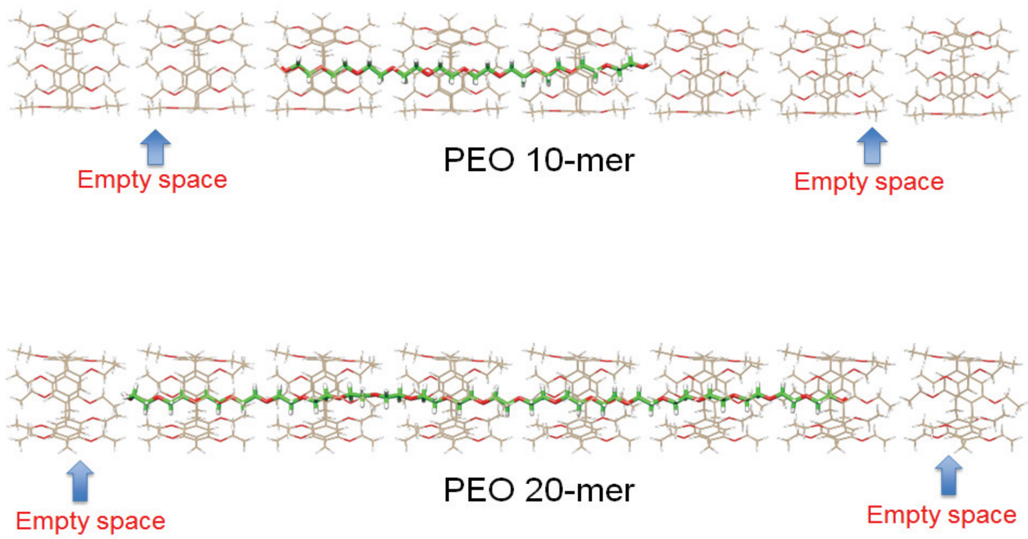

Supplementary Fig. 16 Optimized structures of eight **P5**⊃PEO 10-mer or PEO 20-mer. PEO 10-mer or PEO 20-mer is covered by eight **P5**.

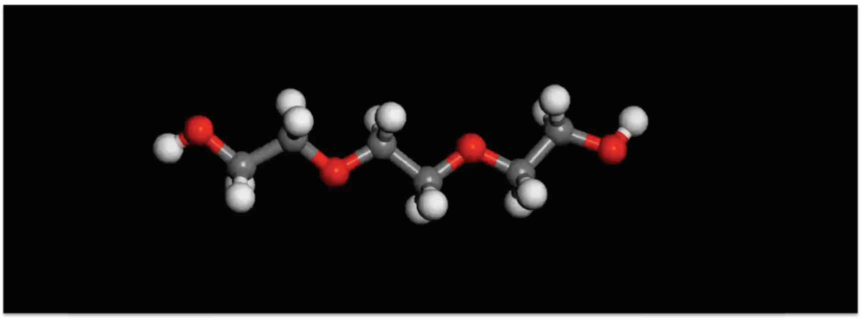

Supplementary Fig. 17 PEO model.

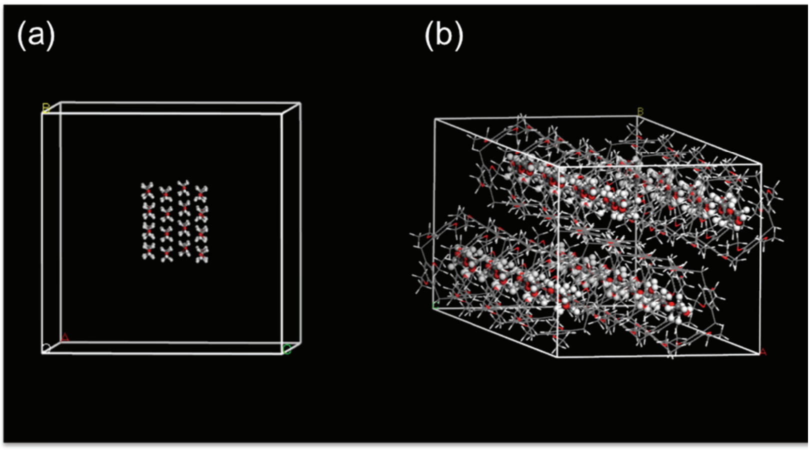

Supplementary Fig. 18 Initial structures for the simulations of (a) free PEO and (b) **P5** ⊃ PEO complex.

(a)

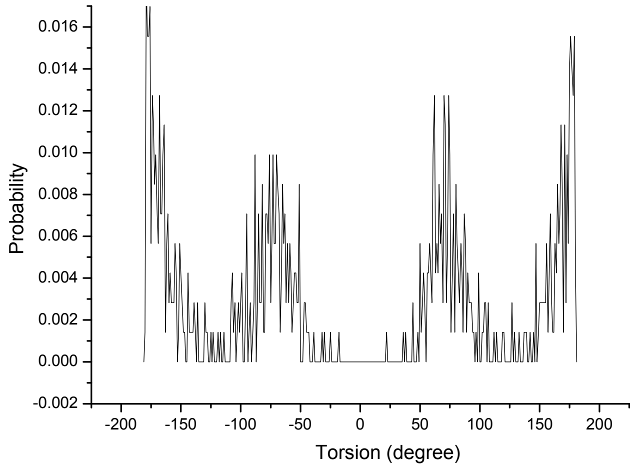

(b)

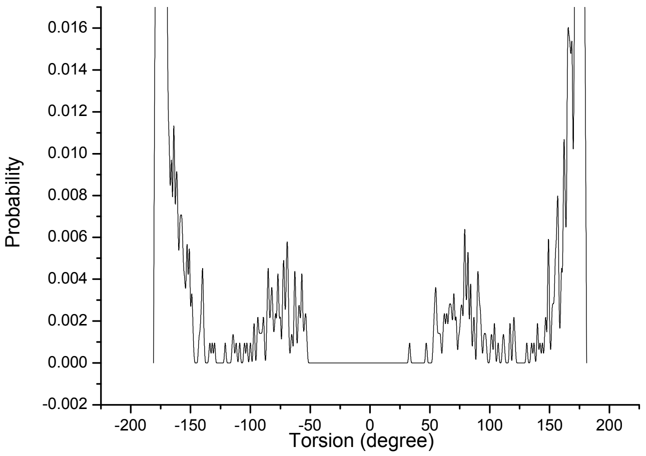

Supplementary Fig. 19. Distributions of torsion angles in the MD trajectories of (a) free PEO and (b) **P5** ⊃ PEO complex.

Supplementary Table 1. Poly(ethylene oxide) (PEO) used in this study

Name	M_w	M_n	Product Number
PEO1000-OH	-	1,000	WAKO165-09085
PEO4000-OH	-	2,700-3,500	Nacalai28221-05
PEO6000-OH	5,400-6,600	-	SERVA39778.01
PEO10000-OH	-	10,000	Aldrich309028-250G
PEO1000-OMe		~1,000	Aldrich445894-50G
PEO1000-NH_2	1,000	-	Alfa Aesar 46833
PEO1000-Ts	-	1,300	Aldrich719080-5G
